# Recurrent Episodes of Thyrotoxicosis in a Man following Pregnancies of his Spouse with Hashimoto's Thyroiditis

**DOI:** 10.1155/2015/940241

**Published:** 2015-09-01

**Authors:** Regina Belokovskaya, Alice C. Levine

**Affiliations:** ^1^Internal Medicine Department, Mount Sinai St. Luke's and Roosevelt Hospitals, Icahn School of Medicine at Mount Sinai, 1111 Amsterdam Avenue, New York, NY 10025, USA; ^2^Division of Endocrinology, Metabolism and Bone Diseases, Icahn School of Medicine at Mount Sinai, 1 Gustave L. Levy Place, P.O. Box 1055, New York, NY 10029, USA

## Abstract

Over an 8-year period, a male patient presented three times to an endocrinologist with strikingly similar presentations, including palpitations, anxiety, and tremors. Each of his presentations occurred following either the birth of one of his two children or his wife's late termination of pregnancy. This patient's illness followed the typical time course of silent thyroiditis: hyperthyroidism, followed by euthyroidism, a late hypothyroid phase, and then a complete resolution of symptoms and normalization of thyroid function tests over a period of several months. We discuss the curious clinical presentation, diagnostic evaluation, and a literature review of alternate explanations for this patient's condition, including a discussion of the impact of seasonal shift, spousal's autoimmune disease, stress, and evolutionary changes in males postpartum. Although the differential diagnosis is broad in this case and the thyrotoxicosis could have coincidentally followed pregnancies of the patient's wife, documented hormonal changes in men during postpartum period in conjunction with the timeline of the patient's condition are suggestive of recurrent “sympathetic” postpartum thyroiditis. To our knowledge, this is the first case report of recurrent painless thyroiditis in a man following pregnancies of his wife with Hashimoto's thyroiditis.

## 1. Introduction

Silent thyroiditis, also known as painless or subacute lymphocytic thyroiditis, is one of several autoimmune thyroid disorders [1]. The clinical presentation of postpartum thyroiditis is virtually identical to that of silent thyroiditis, except that postpartum thyroiditis occurs in women within 1 year after delivery and rarely develops one month postpartum. It is typically associated with the development of either transient hyperthyroidism or hypothyroidism or both up to 1 year postpartum with eventual return to a euthyroid state [2, 3]. Usually, patients have a brief phase of thyrotoxicosis lasting 2–4 weeks, followed by hypothyroidism for 4–12 weeks and then resolution and a return to euthyroidism [4]. In the United States, postpartum thyroiditis occurs in approximately 5–10% of women. The incidence can be greater in certain high-risk populations, including those with a history of autoimmune disorders, with previous thyroid dysfunction including previous postpartum thyroiditis (70% chance of recurrence with subsequent pregnancies), and/or having a family history of thyroid dysfunction [5, 6]. We present a curious case of a man who developed painless thyroiditis three times coinciding with the postpartum period of his wife with Hashimoto's thyroiditis.

## 2. Case Report

A 33-year-old male, with a past medical history of hyperlipidemia, seasonal allergies, and occasional sleep problems, originally presented feeling anxious, hot, and jittery, with recent weight loss and increased frequency of his bowel movements. He appeared slightly anxious, but the rest of exam was unremarkable, including thyroid examination which did not reveal any enlargement, tenderness, or nodules. These symptoms were first reported in 2006 following his wife's late termination of pregnancy performed because of congenital abnormalities noted on prenatal evaluation. He was diagnosed with subacute thyroiditis at that time (no laboratory values are available from his initial presentation) with subsequent normalization of his TSH to the high normal range (4.09, reference range of 0.34–5.60 *μ*IU/Ml) in 2007 and resolution of symptoms. In 2011, several months after his wife's delivery of a healthy baby boy, the patient, who was 38 years old at the time, again presented with symptoms of hyperthyroidism including palpitations, anxiety, and tremors. The TSH was suppressed at 0.07 *μ*IU/Ml (0.34–5.60 *μ*IU/Ml), an elevated free T4 of 3.30 ng/dL (0.60–1.10 ng/dL), free T3 of 9.80 ng/dL (2.50–3.90 ng/dL), thyroglobulin Ab of 448.3 U/Ml (0.0–4.1 U/Ml), and TPO (microsomal Ab) >1000.0 IU/Ml (0.0–5.6 IU/Ml). A markedly decreased thyroid uptake of 0.2% (normal range 10 to 30%) on a nuclear medicine scan was suggestive of the diagnosis of silent thyroiditis. The patient was started on a beta-blocker, propranolol, for symptom control. Several months later, his labs revealed a mildly elevated TSH of 5.73, free T4 of 0.80, free T3 of 3.30, thyroglobulin Ab of 521.1, and TPO of >1000.0 consistent with the hypothyroid phase of thyroiditis. He reported feeling tired, although he no longer experienced heat intolerance or tremors. His weight was stable and his anxiety had significantly subsided. In September 2014, following the birth of his daughter, the patient presented with a similar clinical presentation and a suppressed TSH (0.08 *μ*IU/Ml), elevated free T4 (2.04 ng/dL), elevated free T3 (6.0 ng/dL), thyroglobulin Ab (333.1 U/Ml), and TPO of >1000 IU/Ml. During a follow-up visit, in January 2015, the patient presented in late slightly hypothyroid phase of thyroiditis with TSH of 4.9 *μ*IU/Ml, free T3 of 3.4 Pg/mL, and free T4 of 0.89 NG/DL. TSH from May 2015 is still elevated at 5.0 *μ*IU/Ml and he is now feeling tired and complaining of weight gain. These TFTs were consistent with recurrent painless thyroiditis that coincided with his wife's posttermination and postpartum periods.  Table 1 summarizes these findings.

Of note, the patient's wife did not experience any similar symptoms during or following the three pregnancies. She has a history of Hashimoto's thyroiditis, diagnosed in 2011, when she initially presented with TSH of 96 *μ*IU/Ml, and is maintained on levothyroxine with dose adjustments during gestation.

## 3. Discussion

The case presented here describes recurrent episodes of thyrotoxicosis in a man following pregnancies of his spouse who has treated Hashimoto's thyroiditis. The differential diagnosis for his presentation is broad, and multiple factors might have contributed to this patient's condition. For instance, the thyroid supplementation of the patient's wife suggests that surreptitious ingestion of thyroid hormones is possible. However, the cyclical course of this patient's disease is not consistent with exogenous thyroid hormone use. Given that the recurrent abnormal TFT levels occurred in the summer months, seasonal variations in thyroid levels must be considered, though these are unlikely to be solely responsible for the thyroid hormone variations. The time course of the disease is on the other hand consistent with silent thyroiditis. Patient transitioned from a state of hyperthyroidism to a state of mild hypothyroidism, followed by a resolution of thyroid function tests over a 3-4-month period. Documented hormonal changes in men, during postpartum period, whether stress related or evolutionary in nature, are suggestive of the diagnosis of “sympathetic” postpartum thyroiditis.

Research shows that the environmental impact of living with a spouse suffering from an autoimmune disease should not be ignored. Hemminki et al. looked at epidemiology of Grave's disease and the evidence of familial risks between family members. The study found a high disease concordance of 2.75 between spouses in Swedish population over the course of 20 years, which suggests the operation of some form of environmental sharing. The study mentions similar correlation between spouses and rheumatoid arthritis and amyotrophic lateral sclerosis. Shared smoking habits, psychosocial risk factors, stress, and infections are believed to be the mechanism behind the spouse correlation, all of which may precipitate many autoimmune conditions [7]. Emilsson et al. argued that first-degree relatives and spouses of individuals with celiac disease are at increased risk of nonceliac autoimmune disease. He also suggested that possible sharing of microbiome and the environment with their spouse might impact the risk of developing and diagnosing other autoimmune diseases [8].

Cyclical thyroid hormone changes found in this patient could have been caused by seasonal shifts. In a study about physiological variations in thyroid hormones Fisher states that the levels of T4 are statistically higher in winter as opposed to summer, although the variation is minimal. He attributes this variation to an effect of cold, which accelerates the peripheral metabolism of thyroid hormone during the winter months [9]. A study by Smals et al. found a seasonal variation in circulating serum T4 and T3 levels, inversely correlating with the seasonally altering environmental temperature. Lowest serum T4 and T3 levels were found in the summer [10].

Given that the cycles of thyroid hormone shifts in this patient coincided with his wife's pregnancies, it is important to consider the likelihood of men experiencing similar hormonal changes to women during the postpartum period. In a study by Storey et al. hormone concentrations and responses to infant stimuli in expectant and new fathers living with their partners were measured in order to determine whether men can experience changes that parallel the dramatic shifts seen in pregnant women. The result was that men and women had similar stage-specific differences in hormone levels, including higher concentrations of prolactin and cortisol in the period just before the births and lower postnatal concentrations of testosterone and estradiol. Men reporting pregnancy symptoms and men reporting strong emotional responses to the stimuli had significantly higher prolactin concentrations and experienced a larger testosterone decrease between the two blood samples than men without these responses. The pattern of hormonal change in men demonstrated in this study and its absence in nonpaternal species suggests that hormones may play a role in priming males to provide care for their offspring. From an evolutionary standpoint, it appears that the testosterone decrease in the postnatal period may reduce male's tendencies to engage in nonnurturing behaviors and thus promote greater paternal responsiveness [11].

Considering the similarities in the presentation of autoimmune thyroid diseases, one must often rely on the most common manifestation to determine the diagnosis. For a patient suffering from silent thyroiditis, a presence of ophthalmopathy and/or a thyroid bruit during the thyrotoxic stage would be a strong indication of Grave's disease. Neither presentation was found in this patient. Grave's disease was definitively ruled out by a markedly decreased thyroid uptake on a nuclear medicine scan. Most patients with silent thyroiditis have normal thyroid function at one year. In patients suffering from postpartum thyroiditis, 30 to 50% of patients develop permanent hypothyroidism within nine years [12, 13]. Such appears to be the case with this patient as his TSH levels were found to be elevated during the last two visits. Though the timing of this patient's symptoms is strongly suggestive of postpartum thyroiditis, a causal relationship between episodes of recurrent painless thyroiditis (silent thyroiditis) and spousal postpartum periods has not yet been established.

## 4. Conclusion

The case presented here poses a question whether hormonal changes seen during a postpartum period in a female can manifest themselves in the male spouse. To our knowledge, this is the first case report of a recurrent and possibly “sympathetic” postpartum thyroiditis in a male. Although differential diagnosis is broad, a plausible link between the postpartum period and the coinciding thyrotoxicosis in the male spouse has been presented. A number of documented cases showed that levels of several hormones in men change significantly during their spouse's postpartum period. Such changes, as described, may actually serve an evolutionary purpose. An alternative explanation for our patient's symptoms was established by the inclusion of studies showing a link between living with a spouse suffering from an autoimmune disease and the likelihood of the male partner developing a similar condition. Though more research is required to establish causality, postpartum thyroiditis should be considered as a possible diagnosis for a male patient presenting with anxiety, palpitations, weight loss, and tremors coinciding with postpartum period of his partner.

## Figures and Tables

**Table 1:** Profile of thyroid function tests and timeline of patient's wife postpartum period.

Date	TSH (0.34–5.60 *µ*IU/Ml)	T4, total (5.0–12.2 MCG/DL)	T4, free (0.60–1.10 NG/DL)	T3, free (2.50–3.90 Pg/mL)	Thyroglobulin AB (0.0–4.1 U/mL)	TPO (microsomal) AB (0.0–5.6 IU/mL)
June 2007 (1 year after miscarriage)	4.09	7.2				

September 2007 (1 year and 3 months after miscarriage)	2.78	7.9				

June 2011 (4 months postpartum)	**0.07**		**3.30 **	**9.80**	**448.3**	**>1000.0**

July 2011 (5 months postpartum)	**0.11**		**2.50**	**6.60**		

August 2011 (6 months postpartum)	1.57		**0.40**	2.60		

September 2011 (7 months postpartum)	**5.73**		0.80	3.30	**521.1**	**>1000.0**

June 2012 (1 year and 4 months postpartum)	2.83		0.90	2.98	**139.2**	**237.2**

September 2014 (1 month postpartum, 2nd baby)	**0.08**		**2.04**	**6.00**	**333.1**	**>1000.0**

January 2015 (5 months postpartum, 2nd baby)	4.90		0.89	3.04		
